# Clock/Sleep-Dependent Learning and Memory in Male 3xTg-AD Mice at Advanced Disease Stages and Extrinsic Effects of Huprine X and the Novel Multitarget Agent AVCRI104P3

**DOI:** 10.3390/brainsci11040426

**Published:** 2021-03-26

**Authors:** Lydia Giménez-Llort, Mikel Santana-Santana, Míriam Ratia, Belén Pérez, Pelayo Camps, Diego Muñoz-Torrero, Albert Badia, Maria Victòria Clos

**Affiliations:** 1 Department of Psychiatry and Forensic Medicine & Institut de Neurociències, Universitat Autònoma de Barcelona, E-08193 Barcelona, Spain; mikel.santana@e-campus.uab.cat; 2 Department of Pharmacology, Therapeutics and Toxicology & Institut de Neurociències, Universitat Autònoma de Barcelona, E-08193 Barcelona, Spain; miriam.ratia@e-campus.uab.cat (M.R.); belen.perez@uab.cat (B.P.); albert.badia@uab.cat (A.B.); victoria.clos@uab.cat (M.V.C.); 3 CSIC Associated Unit, Laboratory of Medicinal Chemistry, Faculty of Pharmacy and Food Sciences, Institute of Biomedicine (IBUB), University of Barcelona, E-08028 Barcelona, Spain; camps@ub.edu (P.C.); dmunoztorrero@ub.edu (D.M.-T.)

**Keywords:** sleep, circadian activity, protocols, behavior, drug assessment, aging, Alzheimer’s disease, BPSD, AChEI, multitarget compounds, disease-modifying mechanisms

## Abstract

A new hypothesis highlights sleep-dependent learning/memory consolidation and regards the sleep-wake cycle as a modulator of β-amyloid and tau Alzheimer’s disease (AD) pathologies. Sundowning behavior is a common neuropsychiatric symptom (NPS) associated with dementia. Sleep fragmentation resulting from disturbances in sleep and circadian rhythms in AD may have important consequences on memory processes and exacerbate the other AD-NPS. The present work studied the effect of training time schedules on 12-month-old male 3xTg-AD mice modeling advanced disease stages. Their performance in two paradigms of the Morris water maze for spatial-reference and visual-perceptual learning and memory were found impaired at midday, after 4 h of non-active phase. In contrast, early-morning trained littermates, slowing down from their active phase, exhibited better performance and used goal-directed strategies and non-search navigation described for normal aging. The novel multitarget anticholinesterasic compound AVCRI104P3 (0.6 µmol·kg^−1^, 21 days i.p.) exerted stronger cognitive benefits than its in vitro equipotent dose of AChEI huprine X (0.12 μmol·kg^−1^, 21 days i.p.). Both compounds showed streamlined drug effectiveness, independently of the schedule. Their effects on anxiety-like behaviors were moderate. The results open a question of how time schedules modulate the capacity to respond to task demands and to assess/elucidate new drug effectiveness.

## 1. Introduction

The new hypothesis on the physiological function of sleep highlights sleep-depending learning and memory consolidation [1] and associated plasticity, as well as their strong implications on skill performance involved in many daily life activities [2]. Similarly, visual discrimination learning requires sleep after training [3], and early sleep triggers memory for early visual discrimination skills [4]. Through the life cycle, whereas adequate sleep in childhood and beyond is important for development [5,6], new evidence supports the relevance of the sleep-wake cycle in aging but also since early pre-symptomatic stages of Alzheimer’s disease (AD) [7,8,9,10]. At the pathological level, the sleep-awake cycle controls Aβ levels [11], to the extent that it is regarded as a modulator of AD pathogenesis, while sleep disturbances are proposed as a predictor of dementia and Aβ pathology [12]. The most recent of these outstanding works show that the interstitial fluid and cerebrospinal levels of tau are also regulated by the sleep-wake cycle. Indeed, sleep deprivation increases the levels of tau and the spread of tau pathology [13]. These frequent sleep disturbances, with awakenings during the night and increased proclivity to sleep during daytime [14,15,16], are referred to as sundowning behavior, one of the most common co-morbid clinical manifestations associated with AD. Sleep fragmentation as a result of disturbances in sleep and circadian rhythms in AD may have not only important consequences on memory processes [5,17], but it may also exacerbate the other neuropsychiatric symptoms (NPS) associated with dementia [18], since the impact of sleep in mood disorders, such as anxiety and depression, is also well known and vice versa.

At the translational level, among the different animal models for AD, homozygous 3xTg-AD mice created by LaFerla [19] show a noticeable NPS-like profile, also mimicking diurnal rhythm disturbances [20,21,22,23,24,25]. These animals are sensitive to sleep restriction [26] and display a severe impairment of the functioning of the clock gene pathway [27,28], with males exhibiting worse circadian rhythm disruptions than females [28]. We have recently shown that they have altered slow and fast neocortical oscillations [29]. Therefore, this animal model is useful to investigate whether the alterations of their circadian activity may modify their cognitive performance and the sensitivity of behavioral tests aimed to assess the therapeutic efficacy of new drugs.

Except for those studies of circadian activity, research in the rodents’ natural active dark cycle or using inverted cycle has scarcely been addressed, mostly due to practical reasons. To avoid influences of the time of the day, most behavioral research is usually done during the morning, their non-active circadian period [20]. However, on several occasions, we noticed that cognitive performance of 3xTg-AD mice, but not that of WT mice [30], was sensitive to the time schedules used for training and testing. We hypothesized that early morning was not optimal to observe their impaired cognitive profile in the Morris water maze.

On the other hand, the complexity of AD has led to drug development of multitarget compounds concurrently interfering with different mechanisms, aiming to provide symptomatic relief and exerting disease-modifying benefits [31,32]. In this sense, our multidisciplinary research consortium has synthesized and evaluated the pharmacological profile of new AChEIs, tacrine-huperzine A hybrids, and their derivatives of potential interest for the treatment of AD [33]. Among these, huprines and derivatives have been successfully characterized. Huprine X (HX), a reversible AChE inhibitor hybrid of tacrine and huperzine A, affects the amyloidogenic process in vitro and the AD-related neuropathology in vivo in mice models of AD [34,35,36,37,38,39,40,41,42]. More recently, we have shown that a new family of donepezil-huprine heterodimers that display a dual site binding within AChE has been synthesized, and these compounds have shown inhibitory a highly potent and selective inhibitory action on AChE and BChE. Moreover, they inhibit AChE-induced and self-induced β-amyloid (Aβ) aggregation and β-secretase (BACE-1), and these new drugs are able to cross the blood-brain barrier. Among them, AVCRI104P3 ((±)-3-chloro-12-[(3-{4-[(5,6-dimethoxyindan-2-yl)methyl]piperidin-1-yl}propyl)amino]-6,7,10,11-tetrahydro-9-methyl-7,11-methanocycloocta[*b*]quinoline) has shown an interesting pharmacological profile. It is a potent in vitro inhibitor of human AChE and moderately potent inhibitor of human BChE, AChE-induced, and self-induced Aβ aggregation, and BACE-1 [30].

Therefore, in the present work, we investigated the impact of early morning and midday training schedules on the cognitive performance of male 3xTg-AD mice at 12 months of age, modeling neuropathological advanced disease stages [43]. Since 3xTg-AD mice exhibit circadian rhythm disturbances with sex preference, males were used for the present work purposes [28]. The hypothesis also considered the effect of chronic treatment with anti-Alzheimer compounds [44,45]. AVCRI104P3 is a novel multitarget compound endowed with potent in vitro inhibitory activity of human AChE and moderately potent inhibitory activity of human butyrylcholinesterase (BChE), AChE-induced and self-induced Aβ aggregation, and BACE-1, which has shown behavioral [30,46] and neuroprotective effects [47] in middle-aged mice. For the sake of comparison, we used HX, a potent reversible AChE inhibitor that affects the amyloidogenic process in vitro, and the AD-related neuropathology and behavior in mice models of AD [47]. 

## 2. Materials and Methods

Forty-two 12-month-old homozygous male mice from the Spanish colony of 3xTg-AD mice genetically engineered at the University of California Irvine were used [19]. Subjects were housed in Macrolon cages under standard laboratory conditions of food and water ad libitum, 22 ± 2 °C, 60 ± 10% relative humidity, and a 12/12 h light-dark cycle with lights on at 8:00 a.m. To confirm the findings, two independent experimental sets (March, April) were studied in a counterbalanced manner and blind to the experiment. All animals were treated according to protocols approved by the Department of the Environment and Housing (DMAH, Generalitat de Catalunya, Spain) on 16 March 2014 (certificate No: DMAH-7981). All the research was conducted in compliance with the Spanish legislation on “Protection of Animals Used for Experimental and Other Scientific Purposes” and in accordance with the EU Directive (2010/63/UE) on the NC 3Rs and the efforts to reduce the number of subjects used.

### 2.1. Drug Treatment

AVCRI104P3 ((±)-3-chloro-12-[(3-{4-[(5,6-dimethoxyindan-2-yl)methyl]piperidin-1-yl}propyl)amino]-6,7,10,11-tetrahydro-9-methyl-7,11-methanocycloocta[*b*]quinoline) and HX ((±)-12-amino-3-chloro-9-ethyl-6,7,10,11-tetrahydro-7,11-methanocycloocta[*b*]quinoline) were synthesized as previously described [33,37]. Littermates housed together were distributed into the different groups (*n* = 14/group) and received chronic treatment (i.p., 1 mL/kg) with either HX (0.12 µmol kg^−1^), AVCRIP104P3 (0.6 µmol kg^−1^) or the vehicle (saline) for 21 days, at 3:00 p.m. daily, so that behavioral analysis could be done without interference during the mornings. The chosen doses were those previously used in in vivo studies, shown to exert beneficial effects in middle-aged animals with distinct effectivity at the cognitive and non-cognitive levels [30]. 

### 2.2. Side Effects and NPS-Like Behaviors

Physical condition, weight, and presence/absence of adverse effects, such as diarrhea and tremulous jaw movements, were monitored daily during treatment. Side effects on the locomotor activity were assessed in the classical open-field (OF) test (as compared to basal levels, day 0), as previously detailed [30]. 

### 2.3. Behavioral Assessment

Cognitive performance was assessed in three learning and memory paradigms in the Morris water maze. Furthermore, the same animals were assessed for exploratory activity and anxiety-like behaviors using the dark-light box and corner test, as previously described [30]. 

#### 2.3.1. Exploratory Activity and Anxiety-Like Behaviors

Briefly, locomotor activity, anxiety-like behaviors, and emotionality were assessed by placing the animals in the center of an open-field (woodwork, white, 50 × 50 × 35 cm height, 5 × 5 squares) and observed for 5 min. The latencies to leave the center (central square), reach the peripheral zone (wide square ring next to the walls), and perform the first rearing were noted. Horizontal (number of crossings) and vertical (number of rearings) locomotor activity, the number and duration of grooming, the number of defecations, and urination presence were also recorded. The apparatus was cleaned thoroughly before testing the following animal. Anxiety-like behavior was also measured in the dark/light box. The apparatus (Panlab, S.L., Barcelona, Spain) consisted of two compartments (black, 27 × 18 × 27 cm, white, 27 × 27 × 27 cm, lit with a 20 W white bulb) connected by an opening (7 × 7 cm). The mice were introduced into the black compartment and observed for 5 min. Latency to enter (all four paws) the lit compartment, the time spent in the lit compartment, and the horizontal (3 × 3 squares) and vertical (rearings) activities developed once they were recorded. The apparatus was cleaned thoroughly before testing the following animal. Finally, neophobia to a new home-cage was assessed by introducing the animals into the center of a standard cage filled with 2 L of clean wood cuttings. One cage was used per animal. The number of visited corners and number of rearings were recorded for 30 s.

#### 2.3.2. Morris Water Maze (MWM)

The effects of training and test time schedules as well as those of AVCRI104P3 and HX drugs were assessed from day 15 to 21, after the start of treatment, and compared to saline-treated littermates. In each treatment group, half of the animals were trained to locate a platform (7 cm diameter) in a circular pool (Intex Recreation Corp. CA, USA; 91 cm diameter; 20 cm height, 25 °C opaque water) located in a black test room with distal cues, early in the morning (from 9 to 10 a.m.) and the other half were trained at midday (from 12 to 1 p.m.). Three paradigms were used as previously described [30]: First, we assessed short- and long-term spatial reference learning and memory (6 days, 4 trials 15 min apart, 6 days of place task (PT1-PT6), followed 2 h later by a probe trial for short-term memory) and thereafter, we assessed short-term visual perceptual learning [1 day, 4 trials, 15 min apart, 1 day of cue learning task (CUE)]. Qualitative and quantitative analysis of performance was done by direct observation and analysis of videotape recorded images and considering search and non-search strategies, as described [24,44,45]. 

Days 1–6, Spatial short- and long-term learning and memory (place learning of a hidden platform): This place task (PT) consisted of progressive training of animals to find the platform’s location until all the three experimental groups performed equally. The procedure involved four trial sessions (T1-T4) per day, with trials spaced 15 min apart (short-term memory) during 6 consecutive days (long-term memory). The mouse was gently released (facing the wall) from one starting point randomly selected (N, S, E, or W) and allowed to swim until they located the platform submerged 1.5 cm in a fixed position (SW quadrant and 10 cm away from the wall). The escape latency was recorded. Mice that failed to find the platform within 60 s were placed on it for 10 s, the same period that was allowed for the successful animals.

Day 6, spatial short-term memory (removal): The short-term retention and level of accuracy of the precise location of the platform position achieved were measured in a probe trial or ‘removal’. One and a half hours after the last fourth trial of the place learning, the platform was removed from the maze, and the mice performed a probe trial of 60 s. 

Day 7, visual discrimination perceptual short-term learning and memory (cue learning of a visual platform): In this task, the platform was elevated 1 cm above the water level, with its position in the NW and indicated by a visible striped flag (5 × 8 × 15 cm), whereas external maze cues were removed from the walls. Four trials spaced 15 min apart were performed in one single day. The escape latency was recorded.

Quantitative analyses Behavior was evaluated by both direct observation and analysis of videotape recorded images. Variables of time (escape latency, quadrant preference), distance covered, and swimming speed were analyzed in all the tasks’ trials. The escape latency was readily measured with a stopwatch by an observer unaware of the animal’s genotype and confirmed during the subsequent video-tracking analysis. A video camera placed above the water maze recorded the animal’s behavior, and thereafter, an automated system (Smart, Panlab S.L., Barcelona, Spain) enabled computerized measurement of the distance traveled by the animal during the trials. The swimming speed (cm/s) of the mice during each trial was calculated. In the probe trial, the time spent in each of the four quadrants, the distance traveled along with them and the number of crossings over the removed platform position (annulus crossings) were also measured retrospectively using the automated video-tracking analysis. 

Qualitative analyses Strategy choice in the water maze reveals complex task-solving cognitive paradigms, cognitive flexibility, and spatial learning, but it does not necessarily affect the escape latency or the distance [44]. Therefore, swimming strategies have been extensively characterized by the features of their trajectories mostly based on their goal or non-goal directionality [44]. In the present work, the swim paths for each mouse in each trial of the cue learning task, place learning task, and probe trial were analyzed following the swimming strategies described by Janus [44] and classified according to three criteria: the goal or objective (non-search behaviors, namely floating and circling, vs. search strategies), the direction (goal-directed vs. non-goal directed strategies) and the variety (single vs. mixed strategies) as previously detailed [45].

### 2.4. Statistics

All the analyses were performed according to the SPSS (version 15.0) software. Results are expressed as mean ± standard error of the mean (SEM) or as the incidence of behaviors. Student *t*-test was used to compare differences between two independent groups. Paired *t*-test was used for within-subjects pre-post comparison. In the different Morris water maze tests, the factorial effects of T ‘treatment’, S ‘schedule’, D ’day’, t ‘trial’, and their interactions were analyzed by Split Plot ANOVA for repeated measures followed by post-hoc Bonferroni. Differences in the incidence were measured by Chi-square test. In all cases, statistical significance was considered at *p* < 0.05. 

## 3. Results

### 3.1. Presence of AD-Phenotype before Treatments

Before treatments, the presence of AD-phenotype was confirmed in the open-field test. Thus, naïve 12-month-old 3xTg-AD mice showed poor horizontal (*n* = 42, 44.4 ± 13.3) and vertical (*n* = 42, 9.1 ± 2.8) locomotor activities, as also compared to standard behavior of age-matched NTg mice [30] (vs. *n* = 24, 93.5 ± 14.8, df 64, *p* < 0.01 and vs. *n* = 24, 22.5 ± 4.2, df 64, *p* < 0.05, respectively). Thereafter, animals were distributed counterbalanced in the three treatment groups, with no differences among them in these respects (all, *p* > 0.05) (see Table 1, before treatment).

### 3.2. Absence of Side Effects of AVCRIP104P3 and Huprine X

No side effects, such as tremulous jaw movements, or diarrhea, were observed through treatment. At the end of the treatments, similar weight reduction was found in all the groups (see Table 1, after treatment). When assessed in the open-field, the dark-light, and the corner tests, all animals were found to perform equally, independently of the treatment or assignment in a cognitive training schedule. The repeated OF test resulted in the reduction of exploratory activity, which was stronger in saline-treated animals (*p* = 0.007) with increased urination incidence (*p* = 0.02). AVCRI104P3 and HX-treated mice showed moderated reduction of activity (rearings, 36%, *p* = 0.045 and crossings, 26%, *p* = 0.023, respectively). Increased grooming behavior (*p* = 0.040) was shown in AVCRI104P3-treated mice. 

### 3.3. Training Schedule Affected Learning and Memory

**Figure 1:**
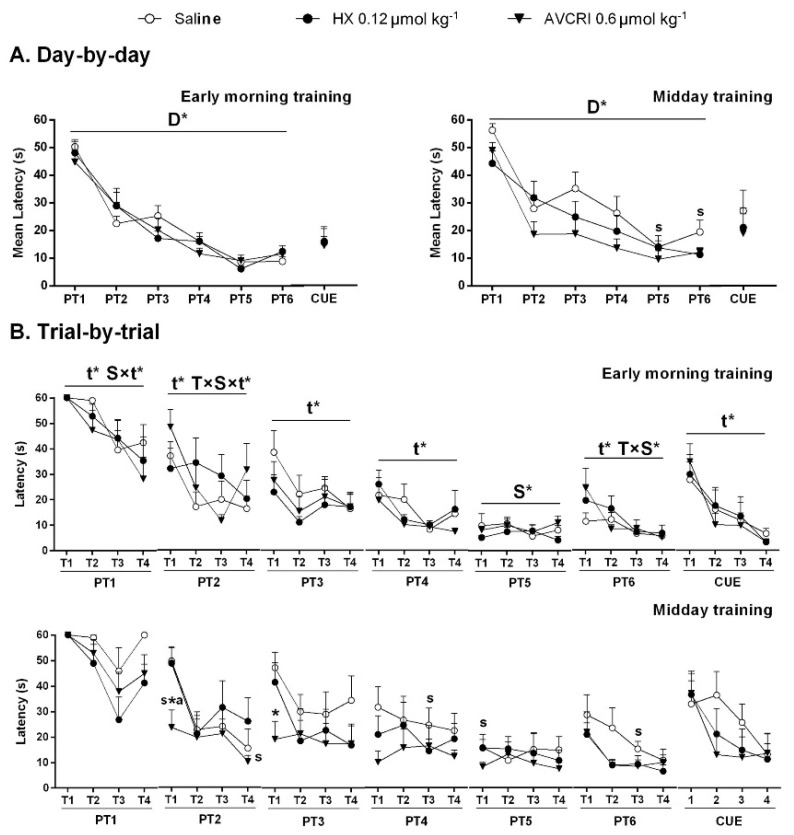
Effects of HX and AVCRIP104P3 on cognitive performance of 3xTg-AD mice in acquiring the place task and cue. Results are expressed as mean ± SEM (*n* = 7 in each group). (**A**) Day-by-day total mean latency to reach a hidden (PTn) and visible (CUE) platform. (**B**) Trial-by-trial latency to reach the platform in each of the four trials (T1–T4) per session. Statistics: Split plot ANOVA: T ‘treatment’, S ‘schedule’, D ‘day’, t ‘trial’, T × S ‘treatment × schedule’, S × t ‘schedule × trial’, T × S × t ‘treatment × schedule × trial’ effects, * *p* < 0.05. Post hoc Bonferroni: * *p* < 0.05 vs. the same schedule saline group, a *p* < 0.05 vs. the same schedule HX group, s *p* < 0.05 vs. the same treatment early morning training group.

The ‘Day-by-day’ analysis (Figure 1A) showed acquisition curves (Day effect, D, *p* < 0.05) in the place and cue learning tasks. Trial-by-trial analysis (Figure 1B) showed trial (Trial effect, t, *p* < 0.05) but also schedule (S) and treatment (T) interaction effects (S × t, T × S × t, S and T × S, all, *p* < 0.05) throughout the intervals of the six days of spatial reference memory (PT1-PT6). 

On their first experience in the MWM (T1, PT1), all groups used up the 60 s trial duration. Only saline-treated 3xTg-AD mice trained at midday invested 60 s in the last trial of that day (T4, PT1). Striking improvement of long-term memory was shown by AVCRIP104P3 mice trained at midday on the next (T1, PT2) and third day (PT3). Thus, in the first 24 h learning and memory trial (PT2, midday schedule), AVCRI104P3 showed a 60% reduction of latency than the first trial performance, while saline and HX groups were equally unsuccessful in finding the platform (mean latency 50 s). Huprine X reached similar optimal performance on day 4 (PT4), and saline did so on day 5 (PT5). Performance in the visual perceptual learning (CUE) was similar among groups and between schedules, with improvements through consecutive repeated trials (T effect, *p* < 0.05).

### 3.4. Training Schedule Affected Memory Retrieval

**Figure 2:**
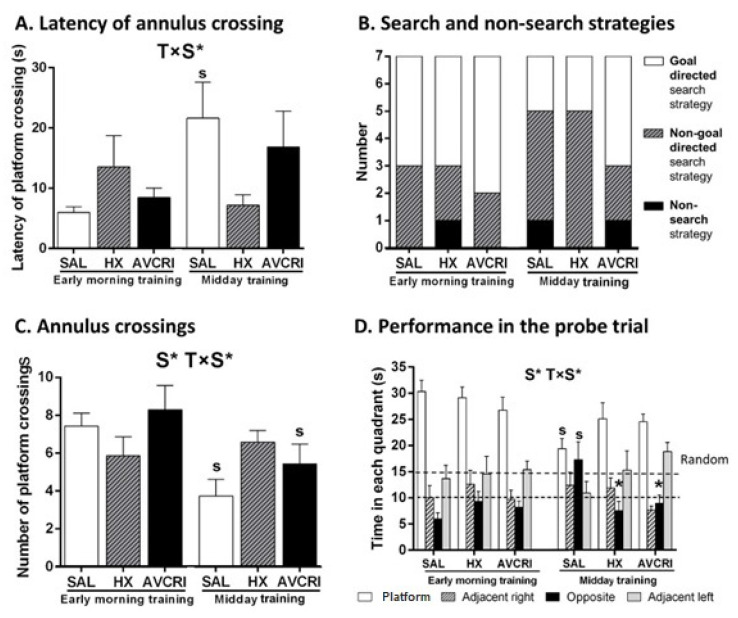
Effects of HX and AVCRIP104P3 on cognitive performance of 3xTg-AD mice in the probe trial for short-term memory in the early morning and midday training schedules. Results are expressed as mean ± SEM (*n* = 7 in each group). (**A**) Latency of platform crossing (s), (**B**) qualitative analysis of search strategies (goal-directed and non-goal directed) and non-search strategies (floating and circling) employed until the achievement of the first annulus crossing, (**C**) annulus crossings, (**D**) time spent in each of the quadrants during the free swim trial: Platform, the trained quadrant where the platform was previously located, adjacent right, opposite and adjacent left quadrants. Time in random preference is indicated with dashed lines. Statistics: Split plot ANOVA: S ‘schedule’ effect, T × S ‘treatment × schedule’ interaction effects, * *p* < 0.05 with post hoc Bonferroni: * *p* < 0.05 vs. the same schedule saline group, s *p* < 0.05 vs. the same treatment early morning training group.

Training schedule affected the probe trial’s performance (S, *p* < 0.0.5), which was better in groups trained early morning. Impaired memory was only shown in saline-treated 3xTg-AD mice trained at midday (T × S interaction effects, *p* < 0.05; post-hoc *p* < 0.05 only for saline). Saline-treated animals showed higher latency of annulus crossing (Figure 2A), used fewer goal-directed strategies, and exhibited non-search strategies (Figure 2B). Lower total number of annulus crossings (Figure 2C) and random search during the whole performance, with absolutely no preference for the trained quadrant (Figure 2D), were also recorded in saline-treated 3xTg-AD mice trained at midday.

Early schedule increased the accuracy of AVCRI104P3-treated mice in their search for the platform, as shown by (1) the number of annulus crossings (Figure 2C) and (2) the quantitative analysis of search and non-search strategies during the probe trial to assess memory (Figure 2B) confirming the higher presence of goal-directed strategies resulting in their improved response in AVCRI104P3-treated mice.

## 4. Discussion

For the first time, the present study shows that the cognitive performance of 12-month-old male 3xTg-AD mice in the MWM was sensitive to the training time schedule, with the expected impaired performance modeling advanced stages of disease only shown in saline-treated animals trained at midday. We also provide first evidence in an AD mouse model of the potent in vivo effects of AVCRIP104P3, a new multitarget drug, as compared to HX. Similar to HX, no side effects were induced by AVCRIP104P3. The streamlined drug effectiveness of AVCRI104P3, with higher potency than its in vitro HX equipotent dose, was stronger at midday. The acquisition improvement was shown in the long-term memory and allowed a faster achievement of optimal performance already on the second day of training.

These results mostly accounted for the place task for spatial reference learning and memory and the subsequent short-term memory performance on the probe trial. There, quadrant preferences and swim strategies used by the experimental groups confirmed different cognitive approaches to solve the paradigm, the AD-cognitive dysfunction clearly manifested in saline-treated animals trained at midday, and the enhanced performance in early morning groups. In the single cue learning task for visual perceptual learning, which also records attentional and motivational aspects, all animals performed equally. However, at midday, a trend to worse performance of saline-treated 3xTg-AD mice suggests that short-term memory was affected, although animals could learn at the end of the day.

Regarding previous work in middle-aged mice, HX was shown to facilitate, in a dose-dependent manner, learning and memory in the MWM [41] using goal-directed accurate search strategies. The benefits were also seen in variables of emotionality and anxiety-like behaviors and the lack of side effects nor affectation of motor activity. Similarly, both ACVRI104P3 (0.06 µmol/kg) and in vitro equipotent dose of HX (0.12 µmol/kg) successfully improved the performance of cognitively poor middle-aged NTg male mice [30]. Both drugs improved short-term learning and memory in a cued task, but ACVRI104P3 also improved both short-term and long-term processes in the MWM and exerted anxiolytic effects in the dark/light box test [30]. Finally, in 12-month-old 3xTg-AD mice, in vitro equipotent doses of HX and HupA improved learning and memory in the MWM, with better acquisition times, faster achievement of improved performance, as well as the presence of goal-directed strategies, and more accurate discrimination of the trained platform in the memory test. They did not induce adverse effects [42]. In the present work, the nootropic effects exerted by HX and ACVRI104P3 in cognitively poor middle-aged mice [30,39] were also confirmed as extensible to middle-aged 3xTg-AD mice. In contrast, the modulation of emotional and anxiety-like behaviors shown in that work only translated to a moderate effect. Both drugs’ benefits were seen independently of the training schedule but more clearly in animals trained at midday. 

It is important to note that the results were observed in two independent experimental sets performed one month apart and that in each set, littermates were distributed in early and midday schedules. Possible involvement of confounding factors was controlled. Thus, animals reared and housed together were distributed into the three treatment groups, and they exhibited similar basal BPSD-levels before treatment. Moreover, the performance of the three groups of mice trained early in the morning was the same, as compared to higher variability among groups at midday. Regarding circadian activity, circadian rhythm disturbances have been reported by several research groups [20,21,22,23,24,25,26,27,28,29], with males better showing these derangements [28]. We reported the overall lowest activity levels of 13-month-old 3xTg-AD mice in their home cage in a 23 h (light and dark) circadian activity compared to age-matched non-transgenic mice. In the particular time frame, from early morning to midday (from 9 a.m. to 1 p.m.), activity levels were normal [24].

In the present work, the training protocol at midday involves sleep disruptions in animals in the fourth hour of their non-active sleeping period. In contrast, the early morning training schedule starts 1 h after the nocturnal activity and involves animals that are still slowing down from their active phase of the circadian cycle [24]. We can hypothesize that the active nocturnal period of rodents, which usually lasts some hours after the switch of the lights, may benefit the level of neuronal activation required to accomplish the task demands. In contrast, once the sleep pattern is achieved, sleep disruption helps to reveal the low cognitive capacity of 3xTg-AD mice, while those animals receiving the anticholinesterasic drugs can counteract these effects. This early sleep-wake cycle may also benefit animals by enhancing the experience-dependent facilitator effect of corticosterone on spatial memory formation in the water maze [47].

The present behavioral evidence meets the growing interest on the crosstalk of the sleep-waves cycle and learning and memory in AD and alerts about the pertinence of taking into account methodological issues relevant to it. The results open a question of how time schedules modulate the capacity to respond to task demands and elucidate new drug effectiveness. Further research should also consider female scenarios, where the lower circadian rhythms disruption may also unveil compensatory mechanisms and modulation by extrinsic pharmacological-related factors.

## Figures and Tables

**Table 1:** Effects of HX and AVCRIP104P3 on BPSD-like behaviors in 3xTg-AD mice at advanced disease stages in each group). Statistics: Student *t*-test, all n.s. *p* > 0.05 vs. the saline group; Paired *t*-test, ^a^ *p* < 0.05, ^aa^ *p* < 0.01vs. before treatment.

	Saline (*n* = 14)	Huprine X 0.12 μmol kg^−1^ (*n* = 14)	AVCRI104P3 0.6 μmol kg^−1^ (*n* = 14)
	Mean ± SEM	Mean ± SEM	Mean ± SEM
Weight			
% vs. initial weight	95.1 ± 1.7	92.8 ± 2.3	93.3 ± 1.5
Open field (before treatment)			
Latency to leave the center (s)	10.79 ± 1.84	11.57 ± 2.03	13.07 ± 2.60
Latency to enter into the periphery (s)	36.50 ± 14.60	32.56 ± 6.48	26.93 ± 5.95
Total number of crossings	46.71 ± 8.62	49.50 ± 10.32	46.71 ± 10.30
Total number of rearings	10.57 ± 2.11	8.29 ± 2.14	9.50 ± 2.03
Number of groomings	2.29 ± 0.40	1.29 ± 0.19	0.71 ± 0.16
Incidence of defecations	13/14	12/14	13/14
Number of defecations	2.29 ± 0.30	2.07 ± 0.37	2.57 ± 0.45
Incidence of urinations	7/14	6/14	6/14
Presence of urine	0.57 ± 0.17	0.50 ± 0.17	0.57 ± 0.23
Open field (after treatment)			
Latency to leave the center (s)	34.50 ± 13.05	13.00 ± 2.97	14.50 ± 1.92
Latency to enter into the periphery (s)	56.64 ± 23.50	39.79 ± 16.97	23.14 ± 4.77
Total number of crossings	32.29 ± 6.86	36.64 ± 8.68 ^a^	36.43 ± 8.29
Total number of rearings	4.43 ± 1.01 ^aa^	5.21 ± 1.43	6.07 ± 1.69 ^a^
Number of groomings	1.14 ± 0.21	1.07 ± 0.20	1.29 ± 0.24 ^a^
Incidence of defecations	13/14	14/14	12/14
Number of defecations	1.86 ± 0.35	2.07 ± 0.29	2.36 ± 0.52
Incidence of urinations	1/14	5/14	2/14
Presence of urine	0.7 ± 0.7 ^a^	0.36 ± 0.13	0.29 ± 0.22
Dark/light box test			
Latency to entry in the lit area (s)	134.29 ± 34.63	140.21 ± 34.05	1.88.29 ± 33.81
Time in the lit area (s)	13.00 ± 3.55	18.79 ± 7.93	15.57 ± 6.28
Number of entries	1.50 ± 0.45	1.93 ± 0.69	1.36 ± 0.43
Total number of risk assessment	3.57 ± 0.84	2.21 ± 0.43	2.43 ± 0.49
Total number of groomings	1.29 ± 0.30	1.36 ± 0.23	2.57 ± 0.91
Incidence of defecations	12/14	11/14	13/14
Number of defecations	2.07 ± 0.37	1.50 ± 0.33	1.79 ± 0.32
Incidence of urinations	6/14	9/14	8/14
Presence of urine	0.43 ± 0.14	1.00 ± 0.43	0.57 ± 0.14
Corner test			
Number of visited corners	5.71 ± 0.87	6.21 ± 0.91	6.36 ± 0.68
Number of rearings	1.64 ± 0.55	3.14 ± 0.64	3.14 ± 0.67

## Data Availability

The data presented in this study are available on request from the corresponding author.
